# 40-year-old Female with Sudden Onset Dyspnea

**DOI:** 10.5811/cpcem.2020.10.49781

**Published:** 2020-12-28

**Authors:** Breanna M. Kebort, Aleta J. Hong, Laura J. Bontempo, Zachary D.W. Dezman

**Affiliations:** * University of Maryland Medical Center, Department of Emergency Medicine, Baltimore, Maryland; † University of Massachusetts Medical School, Department of Emergency Medicine, Worcester, Maryland; ‡ University of Maryland School of Medicine, Department of Emergency Medicine, Baltimore, Maryland

**Keywords:** Dyspnea, adenoid cystic carcinoma, CPC

## Abstract

A 40-year-old female presented to the emergency department (ED) after the acute onset of dyspnea. The patient was tachypneic with accessory muscle usage and diffuse wheezing on initial examination. Despite aggressive treatment, the patient deteriorated and was intubated. This case takes the reader through the differential diagnosis and systematic workup of a patient presenting to the ED with dyspnea and arrives at the unexpected cause for this patient’s presentation.

## CASE PRESENTATION (DR. KEBORT)

A 40-year-old female presented to the emergency department (ED) with sudden onset dyspnea. She stated she had been having progressive shortness of breath over two weeks that worsened acutely just prior to her presentation. The patient also reported a cough productive of yellow/brown mucus over the same time course.

The patient had a past medical history of severe persistent asthma for which she was on multiple inhalers including albuterol sulfate, fluticasone/vilanterol, tiotropium bromide, and ipratropium bromide. She also had a history of seasonal allergies for which she was on dexamethasone nasal spray, fluticasone nasal spray, levocetirizine, ranitidine, and montelukast. She had been using these regularly during her illness with no improvement in symptoms. She recently saw her pulmonologist who found that she had elevated peripheral eosinophils in her blood work and started her on her first injection of mepolizumab.

In addition to asthma, the patient had a medical history of gastroesophageal reflux for which she took pantoprazole, hypertension for which she took losartan, and depression for which she took fluoxetine. She had no surgical history. The patient was a former smoker who had quit nine years earlier. She denied any illicit drug use and reported only social alcohol use. She worked as a nurse at the same location for the prior three years. Her only allergies were to escitalopram and lettuce.

On arrival to the ED, the patient was noted to be in significant respiratory distress with increased work of breathing. She was afebrile (37.5° Celsius) with a heart rate of 135 beats per minute, a respiratory rate of 38 breaths per minute, an oxygen saturation of 100% on room air, and a blood pressure of 160/98 millimeters of mercury. She weighed 88.9 kilograms (kg) and was five feet, five inches in height with a body mass index of 32.6 kg/meter^2^. She was well developed and well nourished. Her head was normocephalic and atraumatic with moist mucous membranes, a clear oropharynx with no uvular edema, tonsillar enlargement or audible stridor. Her neck was supple with no tender lymphadenopathy, and her trachea was midline. She was tachycardic but had a regular rhythm with no murmurs, rubs, or gallops. She was tachypneic and had accessory muscle usage with diffuse wheezing, greatest at the bases. Her abdomen was obese but soft and nontender. She had trace lower extremity edema bilaterally with normal dorsalis pedis pulses. Her lower extremities had no asymmetry or calf tenderness. She had a Glasgow Coma Scale score of 15 with no motor or sensory deficits.

**Image 1:**
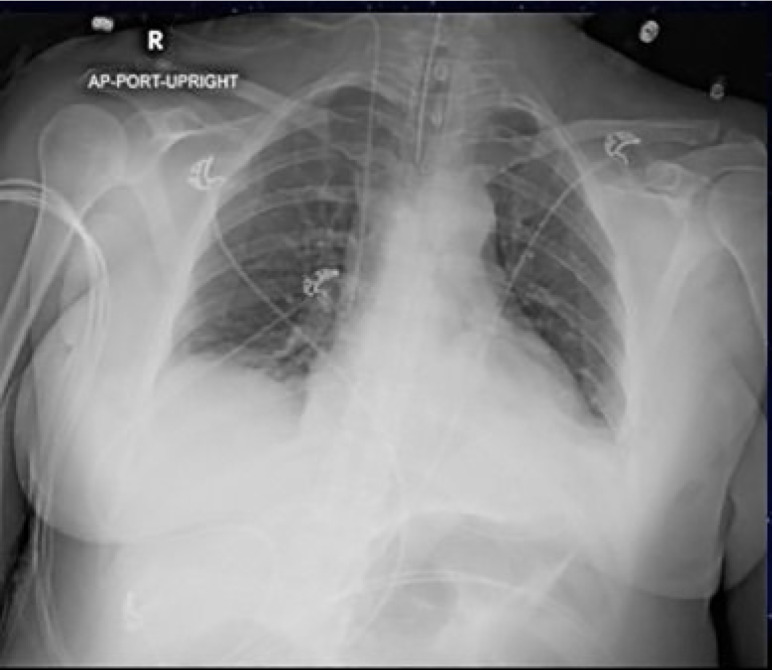
Chest radiograph of a 40-year-old female with sudden onset dyspnea.

An electrocardiogram was performed, which showed sinus tachycardia with a normal axis, normal intervals, and no ST-segment or T-wave changes. Her chest radiography (CXR) is shown (Image 1). Her initial laboratory results are shown in Tables 1 and 2.

The patient received inhaled albuterol 0.083% 5 milligrams (mg) and ipratropium 500 micrograms with 2 grams of intravenous magnesium and solumedrol 125 mg in the ED without significant improvement and with increasing fatigue and declining mental status. Approximately one hour after her arrival to the ED, the patient was intubated using ketamine and rocuronium for presumed status asthmaticus without complication. She was started on broad spectrum antibiotics due to concern for possible pneumonia given her failure to improve. She was admitted to the intensive care unit (ICU). A test was then done that revealed the diagnosis.

## CASE DISCUSSION (DR. HONG)

In this case, a 40-year-old obese female with a history of asthma presented to the ED with difficulty breathing and wheezing. As I read through the case, I was struck by how it served as a valuable reminder that we need to keep our differentials broad, even when the presentation may initially seem clear.

Asthma is a common disease, and as emergency physicians we care for patients with wheezing on a near-daily basis and tend to follow a formulaic treatment pathway, generally with positive results. However, deep down we know that there are more causes to wheezing than just asthma and chronic obstructive pulmonary disease (COPD). It is crucial that whenever we care for a patient who is not improving with standard treatments we step back and reassess our plan, including expanding our differential diagnosis. This is important even when a patient’s presentation seems as though it should be straightforward.

After reading the case presentation, my immediate concern for the patient was her severe respiratory distress complicated by respiratory failure requiring intubation. The physical exam was concerning for impending respiratory failure due to fatigue, and the ED team took the appropriate course of action in intubating the patient prior to decompensation. As emergency clinicians we must never forget the basics of resuscitation and the need to address our ABCs in emergency medicine.

In this case the patient had a history a severe persistent asthma for which she was on multiple medications including short-acting and long-acting beta-agonists; inhaled corticosteroids; montelukast; and she had recently been started on mepolizumab, an interleukin-5 antagonist immunomodulator used in more severe cases of eosinophilic asthma.^1^ Despite her disease severity, a two-week course of symptoms with acute worsening in the setting of medication compliance struck me as unusual. A productive cough combined with minimal response to advanced asthma treatments suggested that her wheezing and respiratory failure was not caused by her asthma alone and that there was another process at play.

At this point I felt I needed to take a step back and reconsider the information I had been presented. Although the patient was noted to be tachypneic on exam with increased work of breathing, her oxygen saturation was 100% on room air and her venous blood gas pH of 7.36 showed no acidosis, both unusual for an asthma exacerbation severe enough to require intubation. Her partial pressure of carbon dioxide (pCO2) was elevated, but the lack of acidosis suggested a more chronic process as the cause of her respiratory failure rather than a severe acute asthma exacerbation. As I considered the many causes of wheezing, I eliminated diagnoses based on the information available.

While there are many causes of wheezing, there are some that could be quickly ruled out based on the patient’s history and physical exam. The physical exam did not note any goiter, or enlargement or asymmetry of the neck. Tonsillar hypertrophy, vascular rings, and bronchiolitis were all unlikely causes of wheezing in an adult. As the patient had no history of recent surgery or intubations and had no history of chronic upper airway inflammation, I reasoned that tracheal stenosis, tracheomalacia, and mucous plugging were unlikely to be the cause of the patient’s wheezing. Vocal cord edema was unlikely as the patient had no stridor or history of voice changes, and, per the ED course she was intubated without any issue.

Given the two-week duration of symptoms and lack of any pertinent history, I removed anaphylaxis from the differential. I also felt that a foreign body was unlikely to be the cause of the patient’s symptoms. While it is possible to have a foreign body present for a prolonged period these are usually small and unlikely to eventually cause acute severe airway compromise. The excellent social history provided by the medical team helped rule out occupational exposures such as bird feces, farming chemicals, or silicosis.

Besides the blood gas, the patient’s laboratory studies did not offer insight into the cause of her symptoms. The CXR was overall unremarkable with the exception of the correct placement of the endotracheal tube. While not diagnostic, I would have expected a patient with respiratory failure due to asthma or COPD to have more hyperinflated lungs than seen on this CXR. There was no sign of a focal infiltrate suggestive of an infectious cause such as bacterial pneumonia or aspergillosis. There were no signs of pulmonary edema on imaging. Given the patient’s lack of history of heart failure I felt comfortable ruling out cardiac causes as the reason for her wheezing. While not a common cause of wheezing, I considered pulmonary embolism (PE) as the patient was tachypneic, tachycardic, and in respiratory distress. Using the revised Geneva score she would be in the moderate risk group, warranting a D-dimer. However, she was neither hypoxic nor hypotensive, unusual for a PE large enough to cause such a significant degree of respiratory failure. Further workup for a PE was therefore not pursued.

At this point I was left with a handful of diagnoses: tumor; autoimmune causes such as eosinophilic granulomatous polyangiitis (EGPA); and infectious causes primarily from pulmonary parasitic infections. While the patient’s history of asthma put her at higher risk for autoimmune diseases like EGPA, she lacked the characteristic extrapulmonary findings such as rash, granulomatous skin lesions, nasal polyps, or recurrent sinusitis.^2^,^3^ Her elevated eosinophil count made pulmonary parasitic infections such as Löffler’s syndrome more likely. Löffler’s syndrome can present insidiously, but the reticulonodular pulmonary opacities typically associated with the disease were not seen on this patient’s CXR. This disease is also rare in the United States, and most of the reported cases are associated with rural living.^4^ All in all, I felt that Löffler’s syndrome would be unlikely.

It can be challenging to narrow a broad differential diagnosis down to a single diagnosis. However, the patient’s smoking history gave me pause as smoking is an independent risk factor for all types of head and neck cancers.^5^,^6^ These cancers often slowly grow in size and can cause both wheezing and severe respiratory distress, depending on their location. After considering a broad differential of potential causes of wheezing, I determined the patient likely had local neck pathology causing an obstruction of the trachea.

If I were the treating physician, I would obtain a computed tomography (CT) of the soft tissue of the neck and chest with intravenous contrast to evaluate for an obstructive mass, most likely in the trachea.

## CASE OUTCOME (DR. KEBORT)

**Image 2:**
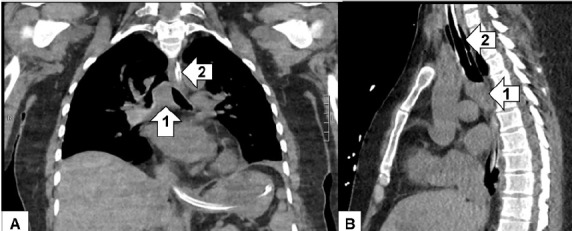
Coronal (A) and sagittal (B) computed tomography of the chest without contrast of a 40-year-old female with dyspnea showing a mass at the carina (1). (Trachea with endotracheal tube [2] is marked to orient the reader.)

**Image 3:**
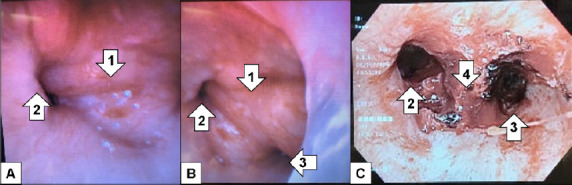
Bronchoscopy images (A, B) of a 40-year-old female with dyspnea showing the carinal mass (1), and the left (2) and right (3) main bronchi. Post-surgical bronchoscopy (C) shows improved cross-sectional diameter of the left (2) and right (3) main bronchi, as well as the area of the resection (4).

After failing to be weaned off the ventilator despite trials of antibiotics, nebulizers, furosemide, terbutaline, and aminophylline, additional imaging was done. The diagnostic study was a CT of the chest. As read by the radiologist, there was a soft tissue mass involving the carina projecting into the lumen of the distal trachea measuring approximately two cubic centimeters (cm) (Image 2) for which direct visualization was recommended. Rigid bronchoscopy showed a large tracheal tumor, which was extensively debulked (Image 3).

The patient was extubated two days after debulking. Her final pathology showed adenoid cystic carcinoma. She had a positron emission tomography (PET) revealing no metastases. Repeat bronchoscopy performed approximately one month later revealed tumor involvement extending two cm from the main carina into the proximal left mainstem bronchus as well as the proximal opening of the bronchus intermedius and into the distal trachea; no lymph nodes sampled were positive for malignant involvement. The patient was deemed to not be a surgical candidate given the extensive airway involvement. She underwent concurrent chemoradiation with weekly carboplatin and paclitaxel as well as proton radiation therapy. Her follow-up CT imaging showed stable irregularity along the tracheal bifurcation with post-radiation changes. She gets repeat CT imaging every three months for continued surveillance.

## RESIDENT DISCUSSION

Primary tracheal tumors are exceedingly rare as the majority of tumors found in the trachea are due to invasion from local tissues including lung, esophagus, or thyroid. In total, primary tracheal tumors account for less than 0.1% of all malignant tumors.^7^ In adults, more than 80% of these primary tracheal tumors are malignant.^8^ Multiple studies show malignant tracheal tumors are more likely to occur in males and usually in the fifth or sixth decades of life.^9^

The most common types of malignant tracheal tumors include squamous cell carcinoma (SCC), adenoid cystic carcinoma (ACC), and carcinoid tumors. Cigarette smoking is a common risk factor for the development of SCC, known to be a fast-growing malignancy. With SCC, the tumor can grow into the mucosa, which can lead to bleeding within the trachea and hemoptysis. ACC has less of a potential to invade the mucosa with bleeding occurring significantly less often.^7^

Despite differences in histology, these malignant tumors present in a similar way with symptoms of airway obstruction being by far the most common. However, these symptoms do not usually occur until at least 50% of the airway is occluded, which can lead to a significant delay in diagnosis. SCC is typically diagnosed within four to six months of initial symptom onset with the most common presentation of hemoptysis, occurring in about 60% of patients with SCC, given its ability to invade the mucosa.^10^ ACC is not diagnosed until an average of 18 months after symptom onset, commonly being misdiagnosed as adult onset asthma due to its overlapping of symptoms of dyspnea on exertion and wheezing.^7^,^8^

The ED workup, as with any patient with new respiratory symptoms, includes a CXR. In addition to identifying lung pathology, the CXR can evaluate for any significant tracheal narrowing. Sensitivity of a CXR is poor with a sensitivity of only 66% when there is a known tracheal mass and can be as low as 20% without a known diagnosis as tracheal tumors can be easily overlooked.^11^ A CT of the chest is usually the next diagnostic step. This can depict the area of obstruction, degree of stenosis, vascular involvement, as well as identify any surrounding lymphadenopathy.^10^

If a tracheal mass is identified on CT imaging, the next diagnostic step is a bronchoscopy for histologic testing and confirmation.^12^ After tissue sampling, PET scans are typically performed for staging of the malignancy although there is no widely accepted staging system in place for tracheal tumors.^8^

Initial management in the ED most importantly included management of the patient’s airway. A smaller-bore endotracheal tube than is typically used in the ED should be selected to prevent a forceful, traumatic airway or inability to pass the endotracheal tube. Steroids do not play a beneficial role in airway obstruction in the setting of tracheal tumors and are therefore not recommended. If a patient had previously been misdiagnosed with asthma and was previously being treated with steroids, these should be discontinued prior to any possible resection.^11^

The main treatment for all types of tracheal malignancies includes resection. However, the maximum length of tissue resection in a trachea is five cm, potentially limiting the full ability to resect it depending on the extent of the tumor. Additional contraindications to surgical resection of these malignancies include multiple confirmed positive lymph nodes, involvement of greater than 50% the length of the trachea, mediastinal invasion of unresectable organs, and distant metastases.^7^ Approximately 50–70% of patients will have resectable disease at their time of diagnosis.^9^ Chemoradiation may be offered in some cases. However, there is a dearth of data regarding its role in treatment of tracheal tumors. At this time, a similar approach is taken to tracheal tumors as those of other tumors with head and neck origin with administration of cisplatin every 21 days along with radiation.^9^ For tumors that are unresectable, rigid bronchoscopy may be performed with laser partial resection or placement of stents, however only as a palliative approach.

The strongest prognostic factor for these patients is the degree of resectability. Squamous cell carcinoma has a worse prognosis with a five-year survival of only 13%. Despite ACC being diagnosed usually significantly later than SCC, with its relatively slow growth and more prolonged course, patients with these tumors have an increased five-year survival of 74%.^13^

## FINAL DIAGNOSIS

Tracheal adenoid cystic carcinoma, complicated by respiratory failure.

## KEY TEACHING POINTS

- When a patient with dyspnea is not improving with standard treatments, reevaluate and reconsider your differential diagnosis.
- Symptoms of tracheal obstruction, such as from a tumor, typically do not present until approximately half of the airway is occluded, which can lead to a delay in diagnosis.
- Initial testing for a tracheal mass includes CXR followed by CT. If a mass is found, bronchoscopy should be performed for direct visualization and tissue sampling.

## Figures and Tables

**Table 1:** Complete blood cell count, serum chemistry and coagulation studies of a 40-year-old female with sudden onset dyspnea.

Blood test	Patient value	Normal range
Complete Blood Count		
White Blood Cells	12.2 K/mcL	4.5 – 11.0 K/mcL
Hemoglobin	11.5 g/dL	12.6 – 17.4 g/dL
Hematocrit	40.4%	37.0 – 50.0%
Platelets	338 K/mcL	153 – 367
Differential		
Polymorphonuclear leukocytes	90.4%	42–75%
Lymphocytes	2.0%	20–50%
Monocytes	6.4%	2–10%
Eosinophils	0.1%	1–3%
Serum chemistries		
Sodium	138 mmol/L	136–145 mmol/L
Potassium	3.8 mmol/L	3.5–5.1 mmol/L
Chloride	104 mmol/L	98–107 mmol/L
Bicarbonate	26 mmol/L	21–30 mmol/L
Blood Urea Nitrogen	10 mg/dL	7–20 mg/dL
Creatinine	0.75 mg/dL	0.66–1.25 mg/dL
Glucose	249 mg/dL	70–99 mg/dL
Magnesium	2.2 mg/dL	1.6–2.6 mg/dL
Phosphorus	3.3 mg/dL	2.5–4.5 mg/dL
Total Protein	5.5 g/dL	6.3–8.2 g/dL
Albumin	3.1 g/dL	3.5–5.2 g/dL
Total Bilirubin	0.3 mg/dL	0.3–1.2 mg/dL
Aspartate Aminotransferase	13 u/L	17–59 u/L
Alanine Aminotransferase	24 u/L	21–72 u/L
Alkaline Phosphatase	49 u/L	38–126 u/L
Lactic Acid	0.9 mmol/L	0.5–1 mmol/L
Coagulation Studies		
Prothrombin Time	13.3 s	10.8–13.3 s
Partial Thromboplastin Time	23.3 s	31.4–48.0 s
International Normalized Ratio	1.1	

*K*, thousand; *mcL*, microliter; *g*, grams; *dL*, deciliter; *mmoL*, millimole; *L*, liter; *mg*, milligrams; *u*, units; *s*, seconds.

**Table 2:** Venous blood gas of 40-year-old female with sudden onset dyspnea.

	Patient value	Normal range
pH	7.30	7.31–7.41
pCO_2_	51 mm Hg	41–51 mm Hg
pO_2_	108 mm Hg	36–42 mm Hg
HCO_3_	29 mmol/L	23–29 mmol/L

*pH,* potential of hydrogen; *mm Hg,* millimeters mercury; *pCO_2_,* partial pressure of carbon dioxide; *pO_2_,* partial pressure of oxygen; *HCO_3_,* bicarbonate.
